# Experiences of a Health System’s Faculty, Staff, and Trainees’ Career Development, Work Culture, and Childcare Needs During the COVID-19 Pandemic

**DOI:** 10.1001/jamanetworkopen.2021.3997

**Published:** 2021-04-02

**Authors:** Rebecca K. Delaney, Amy Locke, Mandy L. Pershing, Claudia Geist, Erin Clouse, Michelle Precourt Debbink, Benjamin Haaland, Amy J. Tanner, Yoshimi Anzai, Angela Fagerlin

**Affiliations:** 1 Department of Population Health Sciences, University of Utah, Salt Lake City; 2 Department of Family and Preventive Medicine, University of Utah Health, Salt Lake City; 3 Department of Sociology, Division of Gender Studies, University of Utah, Salt Lake City; 4 Health Sciences Strategy, University of Utah, Salt Lake City; 5 Department of Obstetrics and Gynecology, University of Utah, Salt Lake City; 6 Office of the Senior Vice President for Health Sciences, University of Utah, Salt Lake City; 7 Department of Radiology, University of Utah, Salt Lake City; 8 Veterans Administration Health Services Research and Development Informatics, Decision-Enhancement and Analytic Sciences Center, Veterans Administration Salt Lake City Health Care System, Salt Lake City, Utah

## Abstract

**Importance:**

In March 2020, US public buildings (including schools) were shut down because of the COVID-19 pandemic, and 42% of US workers resumed their employment duties from home. Some shutdowns remain in place, yet the extent of the needs of US working parents is largely unknown.

**Objective:**

To identify and address the career development, work culture, and childcare needs of faculty, staff, and trainees at an academic medical center during a pandemic.

**Design, Setting, and Participants:**

For this survey study, between August 5 and August 20, 2020, a Qualtrics survey was emailed to all faculty, staff, and trainees at University of Utah Health, an academic health care system that includes multiple hospitals, community clinics, and specialty centers. Participants included 27 700 University of Utah Health faculty, staff, and trainees who received a survey invitation. Data analysis was performed from August to November 2020.

**Main Outcomes and Measures:**

Primary outcomes included experiences of COVID-19 and their associations with career development, work culture, and childcare needs.

**Results:**

A total of 5030 participants completed the entire survey (mean [SD] age, 40 [12] years); 3738 (75%) were women; 4306 (86%) were White or European American; 561 (11%) were Latino or Latina (of any race), Black or African American, American Indian, Alaska Native, and Native Hawaiian or Pacific Islander; and 301 (6%) were Asian or Asian American. Of the participants, 2545 (51%) reported having clinical responsibilities, 2412 (48%) had at least 1 child aged 18 years or younger, 3316 (66%) were staff, 791 (16%) were faculty, and 640 (13%) were trainees. Nearly one-half of parents reported that parenting (1148 participants [49%]) and managing virtual education for children (1171 participants [50%]) were stressors. Across all participants, 1061 (21%) considered leaving the workforce, and 1505 (30%) considered reducing hours. Four hundred forty-nine faculty (55%) and 397 trainees (60%) perceived decreased productivity, and 2334 participants (47%) were worried about COVID-19 impacting their career development, with 421 trainees (64%) being highly concerned.

**Conclusions and Relevance:**

In this survey of 5030 faculty, staff, and trainees of a US health system, many participants with caregiving responsibilities, particularly women, faculty, trainees, and (in a subset of cases) those from racial/ethnic groups that underrepresented in medicine, considered leaving the workforce or reducing hours and were worried about their career development related to the pandemic. It is imperative that medical centers support their employees and trainees during this challenging time.

## Key Points

**Question:**

What are the associations of the COVID-19 pandemic with career development and what are the work culture and childcare needs of employees and trainees?

**Findings:**

In this survey study, most participants with children did not have childcare fully available and many considered leaving the workforce and were worried about their career. Being female with children or having a clinical job role was associated with consideration for leaving the workforce and reducing hours.

**Meaning:**

These findings suggest that a substantial number of employees and trainees experienced major stress and work disruptions because of the COVID-19 pandemic.

## Teaser

This survey study examines the career development, work culture, and childcare needs of faculty, staff, and trainees at an academic medical center during the COVID-19 pandemic,

## Introduction

As a result of the COVID-19 pandemic, 42% of US workers, including many in academic medical centers, were quickly transitioned to working from home in March 2020, and many were simultaneously required to provide childcare and substantial assistance with schoolwork for children during their workdays.^1^ Employed women, in particular, were likely to face greater burdens because they spend 22% more time on unpaid household and care work compared with their male counterparts, with Black or African American and Latina mothers spending nearly twice as much time as men on unpaid housework.^2^ The pandemic has led mothers in heterosexual relationships to reduce their work hours 4 to 5 times more vs fathers because of the pressures of having children at home.^3^ Notably, women comprise 74.9% of hospital employees,^4^ many of whom are essential clinical workers; the extent of the needs and difficulties for these workers during the pandemic remain largely unknown. Employees and trainees who must present to work or training in person may face new childcare expenses for school-age children, resulting in a higher financial burden.

In addition, for those who work in a clinical setting, several studies have shown that there is substantially higher stress for health care workers during the pandemic compared with before the pandemic.^5,6,7,8,9,10^ These life changes are associated with serious concerns about the impact on the careers and well-being of faculty, staff, and trainees. Several recent studies^5^ have examined the effects of burnout, stress, depression, and anxiety on frontline medical staff during the global pandemic. Numerous studies^6,7^ across the globe have demonstrated the substantial burnout of frontline workers, and additional studies^8,9,10^ conducted in the US have shown similar themes. However, many of these studies have been limited by only examining either frontline workers or physician trainees. Most studies also do not address important family-work balance issues, such as childcare needs during the pandemic, which contribute greatly to the stress and burnout of staff.^8^ To our knowledge, no previous studies have examined the work-life needs of both the clinical and nonclinical staff of a medical center.

The primary aim of this survey study was to evaluate the association of the global pandemic caused by SARS-CoV-2 with productivity, career development, and likelihood of leaving the workforce or reducing hours by employees in a tertiary care academic medical center. This study also identified factors (ie, gender, race/ethnicity, job role, and working in a clinical setting) and moderators associated with the aforementioned outcomes. The secondary aim was to describe and identify dependent care and work culture needs to inform institutional policy changes.

## Methods

### Participants

A Qualtrics survey was distributed via email to all 27 700 faculty, staff, and trainees (eg, professional and graduate students, residents, medical fellows, and postdoctoral fellows) at University of Utah Health between August 5 and August 20, 2020, as a quality improvement initiative to inform institutional leadership on how best to support employees. It was sent via a university-wide listserv in which recipients clicked a link to opt into the study anonymously. Because anonymity was imperative to allowing employees to speak freely, there was no system to track differences between responders and nonresponders. The initial email was sent from the CEO and SVP of the University of Utah Health, with 1 reminder email. The University of Utah institutional review board classified the study as exempt and informed consent was waived because the responses were anonymous and because the study presented minimal risk to the participants. Our results follow the American Association for Public Opinion Research (AAPOR) reporting guideline for survey studies.

### Survey Instrument

The survey was developed by a multidisciplinary team of psychologists, physicians, trainees (students, residents, and fellows), and staff. Before launching the survey, a convenience sample of approximately a dozen faculty, staff, and trainees (3-4 people per role) took the survey and provided feedback to identify confusing wording and to suggest additional questions. The survey was mostly quantitative but also included several open-ended questions. The survey took 10 to 20 minutes to complete (contents included in this article are in the eAppendix in the Supplement).

Primary outcome measures were 4-fold, each scored on a Likert scale from 1 (low) to 5 (high). Participants were asked if, as a result of the pandemic, they had considered leaving the workforce, considered reducing their hours, or experienced reduced productivity and whether their career had been impacted. Secondary outcomes included what type of work culture adaptations they would find helpful while working during the pandemic (participants rated their perceived effectiveness of 8 solutions on a 4-point scale from 1 [not helpful at all] to 4 [extremely helpful], with an option for other or not applicable). For those who indicated they had dependent children, additional questions inquired about childcare needs, again with participants rating the effectiveness of potential services (assuming they were provided at an affordable cost) on a 4-point scale from 1 (extremely unlikely) to 4 (extremely likely), with an option for not applicable. Finally, participants completed standard demographic measures. Participants self-reported their race and ethnicity using US Census questions (multiple responses were allowed). Race and ethnicity were measured because we had a priori hypothesized that individuals from groups that are underrepresented in medicine and science would experience the effects of the pandemic differently. For race and ethnicity, we categorized self-reported answers into the following groups for analyses: (1) Asian or Asian American, (2) White or European American, and 3) all racial/ethnic groups that are underrepresented among faculty and trainees, including people who are Hispanic or Latino or Latina of any race, Black or African American, American Indian or Alaskan Native, or Native Hawaiian or Pacific Islander. We separated the aforementioned underrepresented groups from Asian Americans, given that those underrepresented groups are also more likely to be disproportionately affected by adverse health effects of COVID-19.^11^

### Statistical Analysis

Descriptive statistics (proportions or means) are reported for respondent characteristics and primary and secondary outcomes. Four multivariable linear regressions were conducted on the continuous outcome measures. Interaction terms were selected a priori to be entered in the regression analyses. Unstandardized coefficient estimates are presented for regression analyses to represent how much the mean of the dependent variable shifts given 1-unit change in the independent variable (holding other variables in the model constant). To interpret significant moderator effects, the mean estimated value of each outcome was reported for each group.^12^ In the Results section, we discuss the findings of the combined main and interaction effects when interactions are statistically significant and main effects only when interaction terms are not significant. Two-tailed tests were conducted with a significance level of *P* < .05. SPSS statistical software version 26 (IBM) was used to analyze the data. Data analysis was performed from August to November 2020.

## Results

Email invitations to take the survey were sent to 28 000 individuals; 300 emails were returned to sender. Of the remaining 27 700 potential respondents, 5951 (21%) accessed the survey and 5030 (18%) completed the survey and were included in the analysis (mean [SD] age, 40 [12] years). Although more women completed the survey than men (3738 respondents [75%] were women), the proportion is similar to that of women within the health system (across all roles, women typically compose 64% of the workforce, and 74.9% of hospital employees are female).^4^ Respondent characteristics overall and stratified by job role are displayed in Table 1. With regard to race/ethnicity, 4306 (86%) were White or European American; 561 (11%) were Latino or Latina (of any race), Black or African American, American Indian, Alaska Native, and Native Hawaiian or Pacific Islander; and 301 (6%) were Asian or Asian American. Of note, approximately one-half of our sample reported working in a clinical setting (2545 [51%]) and nearly one-half reported having at least 1 child aged 18 years or younger (2412 [48%]). Of the respondents, 3316 (66%) were staff, 791 (16%) were faculty, and 640 (13%) were trainees.

**Table 1.** Characteristics of Survey Respondents

Characteristic	Respondents, No. (%)^a^			
	Overall (N = 4772)	Faculty (n = 791)	Staff (n = 3316)	Trainees (n = 640)
Age, mean (SD), y	39.98 (11.5)	46.56 (10.7)	40.34 (11.2)	29.90 (5.9)
Gender				
No.	5011	834	3494	655
Cisgender female	3738 (75)	505 (61)	2781 (80)	216 (33)
Cisgender male	1158 (23)	309 (37)	628 (18)	430 (66)
Nonbinary, transgender, preferred to self-describe, or preferred not to say	108 (2)	13 (2)	80 (2)	7 (1)
Marital status				
No.	4972	822	3465	653
Married or living with partner	3686 (74)	730 (88)	2512 (73)	424 (65)
Divorced, separated, widowed, or single	1286 (26)	92 (11)	953 (27)	229 (35)
Education level				
No.	4981	825	3474	656
High school degree or less	171 (3)	NA	177 (5)	NA
Some college	686 (14)	NA	627 (18)	57 (8)
2- or 4-y Degree	1851 (36)	10 (1)	1626 (47)	208 (28)
Master's degree	782 (16)	39 (5)	681 (20)	56 (9)
Professional degree	398 (8)	212 (26)	112 (3)	72 (11)
Doctorate	1079 (22)	564 (68)	251 (7)	262 (40)
Race/ethnicity				
No.	5030	827	3500	656
White or European American	4306 (86)	692 (84)	3032 (87)	561 (86)
Hispanic or Latino or Latina of any race, Black or African American, American Indian or Alaskan Native, and Native Hawaiian or Pacific Islander^b^	561 (11)	44 (5)	454 (13)	57 (9)
Asian or Asian American	301 (6)	83 (10)	159 (5)	59 (9)
Work schedule, h/wk				
No.	4622	825	3493	360
≤29	302 (7)	76 (9)	223 (6)	3 (1)
30-39	768 (17)	796(9)	680 (20)	9 (3)
≥40	3552 (77)	673 (81)	2591 (74)	284 (96)
Work in clinical setting				
No.	5030	827	3500	656
No. (%)	2545 (51)	476 (58)^c^	1874 (54)	190 (29)^c^
Have child(ren)				
No.	5030	827	3500	656
No. (%)	2412 (48)	487 (59)	1690 (48)	220 (34)

Abbreviation: NA, not applicable.

^a^ In general, number by job role may differ from overall number because there is missingness for that variable. Not all variables are mutually exclusive (eg, having children), so percentages may not necessarily total 100.

^b^ Categorized together because these races/ethnicities are all underrepresented among faculty, physicians, and trainees.

^c^ Recoded clinical faculty and professional students into clinical category.

### Career Impact and Productivity Among Employees During the COVID-19 Pandemic

Table 2 presents proportions for participants’ consideration of leaving the workforce and reducing hours, work productivity, and worry about the impact of COVID-19 on career development. Overall, 1061 respondents (21%) moderately or very seriously considered leaving the workforce and 1505 (30%) considered reducing hours. Overall, 1359 respondents (27%) felt their productivity increased, whereas 1932 respondents (39%) felt their productivity decreased; 449 faculty (55%) and 397 trainee (60%) respondents showed notably high percentages for reporting decreased productivity. A total of 2334 respondents (47%) reported being moderately or very seriously worried about COVID-19 impacting their career development, with 421 trainee respondents (64%) being highly concerned.

**Table 2.** Participants’ Consideration of Leaving the Workforce or Reducing Hours, Their Work Productivity, and Worry About COVID-19’s Impact on Career Development

Survey response	Participants, No. (%)			
	Overall	Faculty	Staff	Trainees
Moderately or very seriously considered leaving the workforce or educational or training program during the COVID-19 pandemic	1061 (21)	155 (19)	770 (22)	130 (20)
Moderately or very seriously considered reducing hours during the COVID-19 pandemic	1505 (30)	321 (39)	968 (28)	208 (32)
Work or school productivity increased or decreased since the start of the COVID-19 pandemic				
No.	4986	819	3479	653
Decreased	1932 (39)	449 (55)	1074 (31)	397 (60)
Increased	1359 (27)	158 (19)	1088 (31)	99 (15)
Moderately or seriously worried about COVID-19 impacting career development	2334 (47)	377 (46)	1522 (44)	421 (64)
Helpfulness of techniques to manage home and work balance or integration: very or extremely helpful				
Standard meeting time of 50 min rather than 60 min	829 (17)	176 (22)	545 (17)	105 (16)
Designating 12:00-12:30 pm as no-meeting time	1036 (22)	162 (20)	746 (23)	119 (19)
Continued opportunity to work from home after yellow or green COVID-19 levels	2905 (60)	465 (57)	2116 (64)	305 (47)
Flexibility in scheduling meetings, shifts, classes, or clinic time	3276 (68)	532 (66)	2291 (69)	436 (67)
Knowing work or school schedule at least 1 month in advance	2939 (61)	460 (57)	1980 (60)	481 (74)
Ability to turn off video participation in meetings	2286 (48)	375 (46)	1497 (45)	400 (62)
Taking unpaid leave with medical benefits	1748 (37)	207 (26)	1317 (40)	212 (33)
Better understanding of work-life struggles by immediate supervisor	378 (57)	304 (38)	1679 (51)	314 (49)

### Associations of Demographic, Clinical, and Job Role Factors With Career and Productivity Outcomes

Four multivariable linear regression models assessed associations and interactions related to each of the 4 primary outcomes (Table 3). The full regression models are shown in eTable 1, eTable 2, eTable 3, and eTable 4 in the Supplement. Participants who were younger, married, a member of an underrepresented racial/ethnic group, and worked in a clinical setting reported greater consideration for leaving the workforce. Younger, married, and Asian American participants reported a greater consideration for reducing their work hours. Participants who were younger, married, Asian American, male, and had at least 1 child reported decreased productivity, whereas participants who identified as being a member of racial/ethnic group that is underrepresented in medicine reported increased productivity. Younger, Asian American, and underrepresented participants and participants with at least 1 child reported greater worry about the impact of COVID-19 on their career development.

**Table 3.** Four Multivariable Linear Regression Models to Assess Associations and Interactions Related to Four Primary Outcomes^a^

Model and variable	β coefficient (95% CI)			
	Considered leaving workforce	Considered reducing work hours	Reduced work productivity	Worry about impact on career
Model 1				
Age	−0.01 (−0.014 to −0.008)	−0.01 (−0.017 to −0.010)	−0.01 (−0.014 to −0.008)	−0.03 (−0.033 to −0.026)
Female gender	0.21 (0.128 to 0.281)	0.21 (0.119 to 0.297)	−0.16 (−0.234 to −0.084)	−0.12 (−0.208 to −0.043)
Married	0.17 (0.089 to 0.240)	0.38 (0.292 to 0.467)	0.09 (0.019 to 0.167)	−0.01 (−0.098 to 0.065)
Asian	−0.05 (−0.183 to 0.086)	0.16 (0.001 to 0.315)	0.19 (0.054 to 0.319)	0.25 (0.099 to 0.390)
Underrepresented racial/ethnic group	0.11 (0.012 to 0.218)	0.10 (−0.020 to 0.220)	−0.17 (−0.273 to −0.072)	0.13 (0.022 to 0.244)
Model 2				
Clinical job role	0.27 (0.198 to 0.330)	0.27 (0.190 to 0.344)	0.09 (0.029 to 0.156)	−0.10 (−0.172 to −0.029)
Staff	−0.01 (−0.089 to 0.085)	−0.41 (−0.517 to −0.305)	−0.52 (−0.203 to −0.007)	−0.32 (−0.419 to −0.211)
Trainee	−0.10 (−0.236 to 0.027)	−0.41 (−0.562 to −0.257)	0.08 (−0.052 to 0.202)	−0.07 (−0.213 to 0.072)
Model 3				
Has child(ren)	0.37 (0.295 to 0.431)	0.76 (0.685 to 0.838)	0.24 (0.173 to 0.304)	0.32 (0.245 to 0.392)
Model 4				
Gender by have child	0.30 (0.147 to 0.447)	0.40 (0.233 to 0.570)	−0.07 (−0.218 to 0.072)	−0.08 (−0.329 to 0.086)
Gender by staff	0.05 (−0.142 to 0.233)	0.22 (0.011 to 0.433)	0.09 (−0.094 to 0.269)	−0.32 (−0.526 to −0.120)
Gender by trainee	0.27 (0.020 to 0.507)	0.31 (0.033 to 0.581)	0.14 (−0.389 to 0.083)	0.15 (−0.109 to 0.420)
Clinical job by staff	0.15 (−0.028 to 0.320)	0.19 (−0.007 to 0.386)	0.45 (0.278 to 0.616)	0.19 (0.000 to 0.378)
Clinical job by trainee	−0.08 (−0.327 to 0.164)	−0.17 (−0.447)	0.20 (−0.029 to 0.446)	0.25 (−0.017 to 0.516)
Observations, No.	4646	4665	4656	4680

^a^ Given the low sample size for the third gender category (nonbinary, transgender, preferred to self-describe, or preferred not to say), these participants were excluded from this analysis and gender was coded such that 0 = cisgender male and 1 = cisgender female. Other categories were coded as follows: married (0 = single, divorced, separated, widowed, 1 = married or living with partner); Asian (0 = not Asian, 1 = Asian or Asian American); underrepresented ethnic group (0 = not reported underrepresented minority, 1 = Hispanic or Latino or Latina, Black or African American, American Indian or Alaskan Native, Native Hawaiian or Pacific Islander); clinical job role (0 = nonclinical, 1 = clinical); staff (0 = faculty or trainee, 1 = staff); trainee (0 = faculty or staff, 1 = trainee); and has child(ren) (0 = no child, 1 = has child). All of the outcomes are scored 1 (low) to 5 (high).

### Significant Moderators of Association Between Demographic, Clinical, and Job Role Factors With Career and Productivity Outcomes

Statistically significant interactions were found for each of the 4 outcomes (Table 3 and Table 4). The interaction between gender and having dependent child(ren) was significantly associated with consideration for leaving the workforce (β coefficient, 0.30; 95% CI, 0.147-0.447) and reducing hours (β coefficient, 0.40; 95% CI, 0.233-0.570), suggesting that the association between parenthood and career outcomes is different for mothers and fathers. For women, having a dependent child was associated with considerations of leaving the workforce and reducing hours compared with men with a dependent child. Interactions between gender identity and job role were also significant for several of the outcomes. Women in trainee positions considered leaving the workforce (β coefficient, 0.27; 95% CI, 0.020-0.507) and reducing hours (β coefficient, 0.31; 95% CI, 0.033-0.581) more frequently than men in similar positions. For women, faculty or trainee job role was associated with increased worry about the impact of COVID-19 on their career development and greater consideration for reducing hours compared with men in similar roles and women in staff positions. Being a faculty member or trainee with nonclinical responsibilities was also associated with a perceived decrease in productivity and increased worry about the impact of COVID-19 on their career development, compared with nonclinical staff.

**Table 4.** Statistically Significant Interactions by Gender and Clinical vs Nonclinical Job Role

Variable	Likert scale scores, mean (SD)^a^	
Gender	Female	Male
Leaving workforce		
No child	1.58 (0.23)	1.51 (0.26)
Have child	2.06 (0.20)	1.71 (0.17)
Faculty or staff	1.81 (0.31)	1.64 (0.21)
Trainee	1.79 (0.42)	1.50 (0.30)
Reducing hours		
No child	1.68 (0.24)	1.67 (0.21)
Have child	2.61 (0.25)	2.21 (0.37)
Faculty or trainee	2.28 (0.60)	2.12 (0.48)
Staff	2.07 (0.49)	1.81 (0.26)
Faculty or staff	2.12 (0.52)	1.96 (0.41)
Trainee	2.13 (0.59)	1.93 (0.41)
Impact on career development		
Faculty or trainee	2.90 (0.38)	2.67 (0.42)
Staff	2.44 (0.37)	2.65 (0.37)
Work status	Nonclinical	Clinical
Productivity		
Faculty or trainee	2.41 (0.16)	2.59 (0.38)
Staff	3.19 (0.16)	2.97 (0.15)
Impact on career development		
Faculty or trainee	2.90 (0.39)	2.72 (0.42)
Staff	2.47 (0.40)	2.49 (0.36)

^a^ Scores ranged from 1 (low) to 5 (high).

### Dependent Care and Work Culture Needs

Of 2412 participants with children aged 18 years or younger, 1589 (66%) reported that they did not have childcare fully available, and 783 of 2747 (33%) reported being comfortable taking their child to school or a childcare facility (Table 2 and Table 5). Of 2412 participants, 1055 (44%) and 920 (38%) reported that a decrease in COVID-19 cases and the development of a COVID-19 vaccine, respectively, were the most preferred conditions to have to return their child to return to school or childcare. Most participants (1360 participants [67%]) reported they would be somewhat or extremely likely to use temporary services for home childcare (eg, babysitter) or in-person (1231 participants [70%]) and online tutoring (1218 participants [70%]) if they were offered at an affordable cost. A total of 1072 of 2435 participants (45%) felt a lot or a great deal of worry about their ability to provide care and schooling for their children once school started, and 1932 of 2456 participants (81%) reported finding it somewhat or extremely difficult to balance childcare and work responsibilities. Of 2406 participants, 1148 (49%) reported that parenting and 1171 (50%) reported that managing virtual education for children were causing them a lot or a great deal of stress. Of the techniques listed to help manage home and work-balance integration (Table 2), respondents rated the continued opportunity to work from home (2905 participants [60%]), flexibility in scheduling (3276 participants [68%]), knowledge of work-training schedule 1 month in advance (2939 participants [61%]), and a better understanding of work-life struggles by the person they report to (378 participants [57%]) as being very or extremely helpful.

**Table 5.** Participants’ Childcare Needs and Preferences

Variable	Participants, No. (%)			
	Overall (N = 2401)	Faculty (n = 486)	Staff (n = 1681)	Trainees (n = 219)
Children requiring care, mean (SD), No.	1.96 (1.01)	1.99 (.88)	1.95 (1.03)	1.95 (1.05)
Availability of childcare				
No.	2412	487	1690	220
None	581 (24)	125 (26)	405 (24)	47 (21)
Partially available	1008 (42)	202 (42)	717 (42)	80 (36)
Fully available	651 (27)	136 (28)	446 (26)	68 (31)
Not applicable	172 (7)	24 (4.9)	122 (7)	25 (11)
Feel comfortable taking child to care or school if open				
No.	2747	487	1686	220
Yes	783 (33)	177 (36)	527 (31)	75 (34)
No	658 (27)	117 (24)	469 (28)	69 (31)
Uncertain	805 (33)	159 (33)	581 (35)	59 (27)
Not applicable	162 (7)	34 (7)	109 (7)	17 (8)
Preferred conditions to return child to care or school				
No.	2412	487	1690	220
Decrease in cases of COVID-19	1055 (44)	224 (46)	738 (44)	88 (40)
State deems it safe to return to school	286 (12)	48 (10)	213 (13)	22 (10)
Better understanding of how contagious COVID-19 is in kids	676 (28)	124 (26)	493 (29)	57 (26)
COVID-19 vaccine is developed	920 (38)	170 (35)	670 (40)	76 (35)
Risk of losing job	347 (14)	42 (9)	278 (16)	25 (11)
Likelihood of using temporary back-up services during pandemic, if available at an affordable cost				
Childcare in a group setting (ages 0-5 y)	694 (52)	141 (53)	440 (50)	111 (61)
Childcare in a group setting (ages 6-12 y)	516 (39)	124 (44)	354 (38)	35 (40)
Childcare in a group setting (ages 13-18 y)	151 (20)	28 (18)	107 (19)	13 (27)
Childcare in home (eg, babysitter)	1360 (67)	291 (69)	909 (65)	151 (79)
In-person tutoring	1231 (70)	261 (72)	887 (70)	76 (66.7)
Online tutoring	1218 (70)	208 (60)	922 (73)	79 (68)
Help with matching to other families to create small groups of shared childcare, educational instruction, or social interaction (often called pods)	1157 (56)	270 (62)	763 (53)	119 (62)
Help with finding and/or interviewing a babysitter	889 (47)	223 (55)	552 (43)	107 (58)
Consultation with an education specialist on how to better support your children during online learning	1255 (70)	244 (67)	917 (72)	86 (67)
Worried about being able to provide appropriate care or schooling for their children once school starts	1072 (45)	209 (43)	772 (46)	85 (39)
Difficulty balancing childcare and work responsibilities	1932 (81)	422 (89)	1311 (78)	192 (87)
Causes of stress				
Managing distance or online education for children	1148 (49)	222 (47)	857 (51)	63 (29)
Parenting	1171 (50)	222 (46)	833 (50)	115 (53)

## Discussion

In this survey of 5030 faculty, staff, and trainees of a US health system, we found widespread reported stress associated with caregiving, decreased productivity, concerns about career development, and consideration of either reducing hours or leaving the workforce 6 months after the beginning of the COVID-19 pandemic. These experiences were exacerbated for workers who provide clinical care, those with children at home, women, and people of color—especially those who identify as a belonging to a racial/ethnic group that is underrepresented among medical professionals, academics, and trainees. Faculty and trainees (most notably women) and workers with nonclinical job roles in particular reported considering leaving the workforce and reducing hours, experiencing reduced productivity, and facing greater concern about the impact of COVID-19 on their careers.

Given the disproportionate impact COVID-19 has on employees of health systems, institutions must find ways to support their employees, both in terms of workplace cultural adaptations and assistance with familial responsibilities. Most participants indicated they wanted continued flexibility in terms of when and where they work and to receive their work schedules at least 1 month in advance (presumably to make arranging childcare easier). Among workers with children younger than 18 years, we found that although some would like to place their children in temporary childcare center settings, the vast majority preferred assistance in home childcare, tutoring (either in person or virtually), or finding groups of like-minded parents (to form pods).

Although academic centers cannot single-handedly relieve many of the stressors facing employees, they have substantial opportunities to influence the employee experience. Our findings suggest that institutional policies could be developed to support all employees, including families, by addressing telecommuting policies and schedule flexibility, as well as providing expanded support options to address psychological stress of employees and trainees and the educational and direct care needs of their children. The development of new, high-impact stopgap measures, such as tutoring and resource matching, provides an opportunity to meet acute needs related to COVID-19. In addition, because most academic medical centers already offer a variety of services, expanded communication of existing resources can increase access. Finally, the COVID-19 pandemic sheds light on the longstanding difficulties of obtaining affordable childcare, particularly for ill children or when school is unexpectedly closed, providing us with an opportunity to consider longer term needs beyond COVID-19.

### Limitations

Our ability to generalize these data is limited because the survey was sent to employees of only 1 health system; furthermore, the response rate was too low to be generalizable for the entire health system. However, this is one of the first studies to examine the needs of all employees, allowing us to understand the varying needs of different types of employees. Other limitations include selection bias with regard to individuals who chose to complete the survey. Although the email requesting survey completion emphasized the goal for all employees to participate regardless of dependent status, it is possible that more parents of children aged 18 years or younger completed the survey than those without children. The respondents also included a low portion of racial and ethnic groups that are not representative of the US population, although this is mostly accounted for by the overall low population of such groups in the state of Utah. Furthermore, unlike staff and trainees, faculty were not explicitly asked whether they provided clinical care. In our analysis, we included all participants who reported being on the clinical track as providing clinical care, but were unable to categorize physicians who are on tenure track as to whether they provide clinical care. Thus, we may have underreported the percentage of faculty providing clinical care.

## Conclusions

In this survey of 5030 faculty, staff, and trainees of a US health system, our results suggest that respondents were struggling during the COVID-19 pandemic. As a result, even after investing substantial amounts of time in years of training, many were considering leaving the workforce because of stress and caregiving responsibilities related to the pandemic. Health systems must develop effective strategies to ensure that the workplace acknowledges and supports employees during this unprecedented time, not only within the work environment, but also in managing unanticipated childcare responsibilities due to lack of childcare or in-person school. In doing so, health systems will improve the likelihood of retaining generations of well-trained clinicians, scientists, and staff.

## Notes

Supplement. **eAppendix.** Survey Instrument **eTable 1.** Full Regression Model Predicting Participants’ Consideration for Leaving the Workforce (N=4646) **eTable 2.** Full Regression Model Predicting Participants’ Consideration for Reducing Work Hours (N=4642) **eTable 3.** Full Regression Model Predicting Participants’ Reported Work Productivity (N=4634) **eTable 4.** Full Regression Model Predicting Participants’ Reported Concern about COVID-19’s Impact on Career Development (N=4657)

## References

[1] Wong M. Stanford research provides a snapshot of a new working-from-home economy. Stanford News. Published June 29, 2020. Accessed March 3, 2021. https://news.stanford.edu/2020/06/29/snapshot-new-working-home-economy/

[2] Hayes J, Hess C, Ahmed T; Institute for Women's Policy Research. Providing unpaid household and care work in the United States: uncovering inequality. Published January 20, 2020. Accessed March 3, 2021. https://iwpr.org/iwpr-publications/report/providing-unpaid-household-and-care-work-in-the-united-states-uncovering-inequality/

[3] Collins C, Landivar LC, Ruppanner L, Scarborough WJ. COVID-19 and the gender gap in work hours. Gend Work Organ. 2021;28(1)(suppl):101-112. doi:10.1111/gwao.12506 32837019 PMC7361447

[4] Kantamneni N. The impact of the COVID-19 pandemic on marginalized populations in the United States: a research agenda. J Vocat Behav. 2020;119:103439. doi:10.1016/j.jvb.2020.103439 32390658 PMC7205696

[5] Pappa S, Ntella V, Giannakas T, Giannakoulis VG, Papoutsi E, Katsaounou P. Prevalence of depression, anxiety, and insomnia among healthcare workers during the COVID-19 pandemic: a systematic review and meta-analysis. Brain Behav Immun. 2020;88:901-907. doi:10.1016/j.bbi.2020.05.026 32437915 PMC7206431

[6] Azoulay E, Cariou A, Bruneel F, . Symptoms of anxiety, depression, and peritraumatic dissociation in critical care clinicians managing patients with COVID-19: a cross-sectional study. Am J Respir Crit Care Med. 2020;202(10):1388-1398. doi:10.1164/rccm.202006-2568OC 32866409 PMC7667906

[7] Luceño-Moreno L, Talavera-Velasco B, García-Albuerne Y, Martín-García J. Symptoms of posttraumatic stress, anxiety, depression, levels of resilience and burnout in Spanish health personnel during the COVID-19 pandemic. Int J Environ Res Public Health. 2020;17(15):5514. doi:10.3390/ijerph17155514 32751624 PMC7432016

[8] Kannampallil TG, Goss CW, Evanoff BA, Strickland JR, McAlister RP, Duncan J. Exposure to COVID-19 patients increases physician trainee stress and burnout. PLoS One. 2020;15(8):e0237301. doi:10.1371/journal.pone.0237301 32760131 PMC7410237

[9] Khalafallah AM, Lam S, Gami A, . A national survey on the impact of the COVID-19 pandemic upon burnout and career satisfaction among neurosurgery residents. J Clin Neurosci. 2020;80:137-142. doi:10.1016/j.jocn.2020.08.012 33099336 PMC7438065

[10] Shechter A, Diaz F, Moise N, . Psychological distress, coping behaviors, and preferences for support among New York healthcare workers during the COVID-19 pandemic. Gen Hosp Psychiatry. 2020;66:1-8. doi:10.1016/j.genhosppsych.2020.06.007 32590254 PMC7297159

[11] Moore J, Ricaldi JN, Rose CE, . Disparities in incidence of COVID-19 among underrepresented racial/ethnic groups in counties identified as hotspots during June 5-18, 2020: 22 states, February-June 2020. MMWR Morb Mortal Wkly Rep. 2020;69:1122-1126. doi:10.15585/mmwr.mm6933e1 32817602 PMC7439982

[12] Frazier PA, Tix, AP, Barron, KE. Testing moderator and mediator effects in counseling psychology research. J Counsel Psychol Res. 2004;51(1):115-134. doi:10.1037/0022-0167.51.1.115

