# Supplementary material for: Experiences of a Health System’s Faculty, Staff, and Trainees’ Career Development, Work Culture, and Childcare Needs During the COVID-19 Pandemic
Source: JAMA Netw Open. 2021 Apr 2;4(4):e213997. doi: 10.1001/jamanetworkopen.2021.3997 (PMC8019096; doi:10.1001/jamanetworkopen.2021.3997)
Supplement: Supplement. — eAppendix. Survey Instrument eTable 1. Full Regression Model Predicting Participants’ Consideration for Leaving the Workforce (N=4646) eTable 2. Full Regression Model Predicting Participants’ Consideration for Reducing Work Hours (N=4642) eTable 3. Full Regression Model Predicting Participants’ Reported Work Productivity (N=4634) eTable 4. Full Regression Model Predicting Participants’ Reported Concern about COVID-19’s Impact on Career Development (N=4657) [file jamanetwopen-e213997-s001.pdf]

Supplemental Online Content

Delaney RK, Locke A, Pershing ML, et al. Experiences of a health system's faculty, staff, and trainees' career development, work culture, and childcare needs during the COVID-19 pandemic. *JAMA Netw Open*. 2021;4(4):e213997. doi:10.1001/jamanetworkopen.2021.3997

eAppendix. Survey Instrument
eTable 1. Full Regression Model Predicting Participants' Consideration for Leaving the Workforce (N=4646)
eTable 2. Full Regression Model Predicting Participants' Consideration for Reducing Work Hours (N=4642)
eTable 3. Full Regression Model Predicting Participants' Reported Work Productivity (N=4634)
eTable 4. Full Regression Model Predicting Participants' Reported Concern about COVID-19's Impact on Career Development (N=4657)

This supplemental material has been provided by the authors to give readers additional information about their work.

© 2021 Delaney RK et al. *JAMA Network Open.*

eAppendix: Survey Instrument

Note: This is an abridged version of the original survey; only questions that pertain to the present research are shared for the sake of brevity.

Part 1: Introduction

1. We understand that the COVID-19 pandemic is a challenging time for our community, nation, and across the globe. We would like to be able to support our U of U Health employees during this difficult time. There are various options examined in this survey, the feasibility of which will be determined by the survey's results and other factors.

   This survey is anonymous. It is designed to help us understand the dependent care needs of our faculty, staff, and trainees during the COVID-19 pandemic. We will use this information to propose how to best help families in the coming year.

   Thank you for taking our survey.

2. Do you have dependents at home (children or adults living with you who need your care) or elderly parents outside your home who need care or support? (Check all that apply)
   (1) Yes, have children who need care
   (2) Yes, have adults who need care
   (3) No

[For those who indicate that they have children who need care, Child Care block is displayed]

Part 2: Child Care Block

1. How many children do you have that require some level of care? (1 – 25)

2. Is your child care center, school, or babysitting arrangement still currently available to you now in person?
   (1) Fully available
   (2) Partially available
   (3) No
   (4) Not applicable

3. If your childcare center/school was open, would you feel comfortable taking your children to childcare/school?
   (1) Yes
   (2) No
   (3) Uncertain
   (4) Not applicable

4. What conditions would need to exist for you to return to a child care center/school? (Check all that apply)
   (1) Decrease in cases of COVID-19

© 2021 Delaney RK et al. *JAMA Network Open.*

(2) If the State deems it is safe for children to return to school
(3) Better understanding of how contagious COVID-19 is in kids
(4) COVID-19 vaccine is developed
(5) Risk of losing job
(6) Other (please describe)

5. Below are a number of potential, temporary back-up services that may be helpful for those with child care needs during the COVID-19 pandemic. How likely would you be to use each of the following services, if available at what you consider an affordable cost?

© 2021 Delaney RK et al. *JAMA Network Open.*

	Not applicable	Extremely unlikely	Somewhat unlikely	Somewhat likely	Extremely likely
Child care in a group setting for ages 0-5	◯	◯	◯	◯	◯
Child care in a group setting for ages 6-12	◯	◯	◯	◯	◯
Child care in a group setting for ages 13-18	◯	◯	◯	◯	◯
Child care in home (e.g., babysitter)	◯	◯	◯	◯	◯
In-person tutoring	◯	◯	◯	◯	◯
Online tutoring	◯	◯	◯	◯	◯
Help with matching to other families to create small groups of shared childcare/educational instruction/social interaction (often called pods)	◯	◯	◯	◯	◯
Help with finding and/or interviewing a babysitter	◯	◯	◯	◯	◯

© 2021 Delaney RK et al. *JAMA Network Open.*

Consultation with an education specialist on how to better support your children during online learning	○	○	○	○	○
Other (please describe):	○	○	○	○	○

6. How worried are you that you would not be financially able to take advantage of the services you indicated interest in?
   (1) Not at all
   (2) A little
   (3) A moderate amount
   (4) A lot
   (5) A great deal

7. How difficult have you found balancing child care and work responsibilities?
   (1) Extremely difficult
   (2) Somewhat difficult
   (3) Neither easy nor difficult
   (4) Somewhat easy
   (5) Extremely easy

[End of block: Child Care]

© 2021 Delaney RK et al. *JAMA Network Open.*

Part 3: All Participants Block

1. Have you considered leaving the workforce or your educational/training program during the COVID-19 pandemic?
   (1) Haven't considered at all
   (2) Considered slightly
   (3) Moderately considered
   (4) Seriously considered
   (5) Very seriously considered

2. Have you considered reducing your hours during the COVID-19 pandemic?
   (1) Haven't considered at all
   (2) Considered slightly
   (3) Moderately considered
   (4) Seriously considered
   (5) Very seriously considered

3. [If have child dependents] How much is the following causing you stress?

	None at all	A little	A moderate amount	A lot	A great deal
Managing distance/online education for children	○	○	○	○	○
Parenting	○	○	○	○	○

4. Has your work/school productivity increased or decreased since the start of the COVID-19 pandemic?
   (1) Decreased a lot
   (2) Decreased somewhat
   (3) About the same
   (4) Increased somewhat
   (5) Increased a lot

5. How worried are you that COVID-19 is impacting your career development?
   (1) Not at all
   (2) A little
   (3) A moderate amount

© 2021 Delaney RK et al. *JAMA Network Open.*

(4) A lot
(5) A great deal

6. How helpful would the following techniques be for managing home and work balance or integration?

	Not helpful at all	Slightly helpful	Moderately helpful	Very helpful	Extremely helpful
Standard meeting time of 50 rather than 60 minutes	◯	◯	◯	◯	◯
Designating 12-12:30 pm as a no-meeting time	◯	◯	◯	◯	◯
Continued opportunity to work from home after yellow or green COVID levels	◯	◯	◯	◯	◯
Flexibility in scheduling meetings, shifts, classes, or clinic time	◯	◯	◯	◯	◯
Knowing my work/school schedule at least one month in advance	◯	◯	◯	◯	◯
Ability to turn off video participation in meetings	◯	◯	◯	◯	◯
Taking unpaid leave with medical benefits	◯	◯	◯	◯	◯

© 2021 Delaney RK et al. *JAMA Network Open.*

Better understanding of work-life struggles by the person(s) you report to	○	○	○	○	○
Other (Please explain):	○	○	○	○	○

Part 4: Demographics

1. What is your gender?
   (1) Female
   (2) Male
   (3) Transgender woman / transwoman
   (4) Transgender man / transman
   (5) Non-binary / third gender
   (6) Prefer to self-describe
   (7) Prefer not to say

2. What is your marital status?
   (1) Single
   (2) Married / living with partner
   (3) Widowed
   (4) Divorced
   (5) Separated

3. What is your age? (18 – 99)

4. What is your education level?
   (1) Less than high school
   (2) High school graduate
   (3) Some college
   (4) 2 year degree
   (5) 4 year degree
   (6) Master’s degree
   (7) Professional degree
   (8) Doctorate

© 2021 Delaney RK et al. *JAMA Network Open.*

5. How would you describe your race? (Mark all that apply)
   (1) American Indian or Alaska Native
   (2) Asian or Asian American
   (3) Black or African American
   (4) Native Hawaiian or other Pacific Islander
   (5) White or European American
   (6) Other (please specify)

6. Are you Hispanic or Latino/a or Latinx?
   (1) Yes
   (2) No

7. What is your role in the University?
   (1) Faculty
   (2) Staff
   (3) Student
   (4) Resident
   (5) Medical fellow
   (6) Post-doctoral fellow

8. [If staff] Does your job require you to work in a clinical setting (e.g., provide direct patient clinical care, provide environmental services)?
   (1) Yes
   (2) No

9. [If not a student] How many hours a week do you work in your UofU position?
   (1) 0 – 9 hours per week (up to .24 FTE)
   (2) 10 – 19 hours per week (0.25 – 0.5 FTE)
   (3) 20 – 29 hours per week (0.5 – 0.75 FTE)
   (4) 30 – 39 hours per week (0.75 – 0.99 FTE)
   (5) 40+ hours per week (1.00 FTE)

© 2021 Delaney RK et al. *JAMA Network Open.*

eTable 1. Full Regression Model Predicting Participants' Consideration for Leaving the Workforce (N=4646)

Model	R	R Square	Adjusted R Square	Std. Error of the Estimate	Change Statistics				
					R Square Change	F Change	df1	df2	Sig. F Change
1	.144[a]	0.021	0.020	1.124	0.021	19.701	5	4640	0.000
2	.190[b]	0.036	0.035	1.115	0.015	24.856	3	4637	0.000
3	.242[c]	0.059	0.057	1.102	0.022	109.408	1	4636	0.000
4	.252[d]	0.064	0.061	1.100	0.005	5.009	5	4631	0.000

**ANOVA[a]**						
		Sum of Squares	df	Mean Square	F	Sig.
Model 1	Regression	124.343	5	24.869	19.701	.000[b]
	Residual	5856.992	4640	1.262		
	Total	5981.335	4645			
Model 2	Regression	217.038	8	27.130	21.824	.000[c]
	Residual	5764.297	4637	1.243		
	Total	5981.335	4645			
Model 3	Regression	349.937	9	38.882	32.009	.000[d]
	Residual	5631.398	4636	1.215		
	Total	5981.335	4645			
Model 4	Regression	380.227	14	27.159	22.455	.000[e]
	Residual	5601.108	4631	1.209		
	Total	5981.335	4645			

© 2021 Delaney RK et al. *JAMA Network Open.*

**Coefficients[a]**								
		Unstandardized Coefficients		Standardized Coefficients	t	Sig.	95.0% Confidence Interval for B	
		B	Std. Error	Beta			Lower Bound	Upper Bound
Model 1		1.903	0.073		26.012	0.000	1.760	2.046
	Age	-0.011	0.001	-0.108	-7.313	0.000	-0.014	-0.008
	Female	0.205	0.039	0.077	5.253	0.000	0.128	0.281
	Married	0.164	0.039	0.063	4.267	0.000	0.089	0.240
	Asian	-0.049	0.069	-0.010	-0.708	0.479	-0.183	0.086
	Under Represented Minorities	0.115	0.053	0.032	2.184	0.029	0.012	0.218
Model 2		1.815	0.099		18.310	0.000	1.620	2.009
	Age	-0.011	0.002	-0.109	-6.787	0.000	-0.014	-0.008
	Female	0.181	0.039	0.068	4.587	0.000	0.103	0.258
	Married	0.161	0.039	0.062	4.175	0.000	0.085	0.236
	Asian	-0.026	0.069	-0.005	-0.376	0.707	-0.160	0.109
	Under Represented Minorities	0.094	0.052	0.026	1.791	0.073	-0.009	0.197
	Clinical job role	0.264	0.034	0.116	7.816	0.000	0.198	0.330
	Staff	-0.007	0.047	-0.003	-0.141	0.888	-0.098	0.085
	Trainee	-0.104	0.067	-0.032	-1.557	0.120	-0.236	0.027
Model 3		1.544	0.101		15.242	0.000	1.346	1.743

© 2021 Delaney RK et al. *JAMA Network Open.*

	Age	-0.007	0.002	-0.075	-4.630	0.000	-0.011	-0.004
	Female	0.189	0.039	0.071	4.863	0.000	0.113	0.266
	Married	0.046	0.040	0.018	1.166	0.244	-0.031	0.124
	Asian	-0.032	0.068	-0.007	-0.471	0.638	-0.165	0.101
	Under Represented Minorities	0.075	0.052	0.021	1.440	0.150	-0.027	0.176
	Clinical job role	0.253	0.033	0.111	7.566	0.000	0.187	0.318
	Staff	0.033	0.046	0.014	0.721	0.471	-0.057	0.124
	Trainee	0.018	0.067	0.005	0.268	0.789	-0.114	0.150
	Has Child(ren)	0.363	0.035	0.160	10.460	0.000	0.295	0.431
Model 4		1.757	0.123		14.240	0.000	1.515	1.999
	Age	-0.007	0.002	-0.074	-4.577	0.000	-0.011	-0.004
	Female	-0.037	0.093	-0.014	-0.398	0.691	-0.220	0.146
	Married	0.046	0.040	0.018	1.161	0.246	-0.032	0.123
	Asian	-0.030	0.068	-0.006	-0.436	0.663	-0.162	0.103
	Under Represented Minorities	0.066	0.052	0.019	1.282	0.200	-0.035	0.168
	Clinical job role	0.158	0.080	0.070	1.966	0.049	0.000	0.316
	Staff	-0.070	0.093	-0.028	-0.744	0.457	-0.253	0.114
	Trainee	-0.150	0.117	-0.045	-1.288	0.198	-0.379	0.078
	Has Child(ren)	0.142	0.068	0.062	2.096	0.036	0.009	0.274
	Gender by Have Child	0.297	0.076	0.126	3.881	0.000	0.147	0.447

© 2021 Delaney RK et al. *JAMA Network Open.*

	Gender by Staff	0.045	0.096	0.020	0.475	0.635	-0.142	0.233
	Gender by Trainee	0.263	0.124	0.066	2.118	0.034	0.020	0.507
	Clinical job by Staff	0.146	0.089	0.062	1.640	0.101	-0.028	0.320
	Clinical job by Trainee	-0.081	0.125	-0.014	-0.649	0.516	-0.327	0.164

© 2021 Delaney RK et al. *JAMA Network Open.*

eTable 2. Full Regression Model Predicting Participants' Consideration for Reducing Work Hours (N=4642)

**Model Summary**									
Model	R	R Square	Adjusted R Square	Std. Error of the Estimate	Change Statistics				
					R Square Change	F Change	df1	df2	Sig. F Change
1	.175[a]	0.031	0.029	1.307	0.031	29.183	5	4636	0.000
2	.234[b]	0.055	0.053	1.291	0.024	39.818	3	4633	0.000
3	.356[c]	0.126	0.125	1.242	0.072	379.545	1	4632	0.000
4	.365[d]	0.134	0.131	1.237	0.007	7.563	5	4627	0.000

**ANOVA[a]**						
		Sum of Squares	df	Mean Square	F	Sig.
Model 1	Regression	249.394	5	49.879	29.183	.000[b]
	Residual	7923.826	4636	1.709		
	Total	8173.219	4641			
Model 2	Regression	448.560	8	56.070	33.629	.000[c]
	Residual	7724.659	4633	1.667		
	Total	8173.219	4641			
Model 3	Regression	1033.581	9	114.842	74.507	.000[d]
	Residual	7139.638	4632	1.541		
	Total	8173.219	4641			
Model 4	Regression	1091.455	14	77.961	50.937	.000[e]
	Residual	7081.764	4627	1.531		
	Total	8173.219	4641			

© 2021 Delaney RK et al. *JAMA Network Open.*

Coefficients[a]								
		Unstandardized Coefficients		Standardized Coefficients	t	Sig.	95.0% Confidence Interval for B	
		B	Std. Error	Beta			Lower Bound	Upper Bound
Model 1	(Constant)	2.168	0.085		25.475	0.000	2.002	2.335
	Age	-0.014	0.002	-0.119	-8.104	0.000	-0.017	-0.010
	Female	0.208	0.045	0.067	4.590	0.000	0.119	0.297
	Married	0.380	0.045	0.124	8.462	0.000	0.292	0.467
	Asian	0.158	0.080	0.029	1.974	0.048	0.001	0.315
	Under Represented Minorities	0.100	0.061	0.024	1.641	0.101	-0.020	0.220
Model 2		2.472	0.115		21.526	0.000	2.247	2.697
	Age	-0.016	0.002	-0.139	-8.680	0.000	-0.020	-0.012
	Female	0.235	0.046	0.075	5.147	0.000	0.145	0.324
	Married	0.344	0.045	0.113	7.708	0.000	0.257	0.432
	Asian	0.127	0.079	0.023	1.605	0.109	-0.028	0.283
	Under Represented Minorities	0.109	0.061	0.026	1.796	0.073	-0.010	0.228
	Clinical job role	0.267	0.039	0.101	6.814	0.000	0.190	0.344
	Staff	-0.411	0.054	-0.143	-7.600	0.000	-0.517	-0.305
	Trainee	-0.410	0.078	-0.106	-5.268	0.000	-0.562	-0.257

© 2021 Delaney RK et al. *JAMA Network Open.*

Model 3		1.904	0.114		16.674	0.000	1.680	2.128
	Age	-0.009	0.002	-0.077	-4.920	0.000	-0.012	-0.005
	Female	0.253	0.044	0.081	5.768	0.000	0.167	0.339
	Married	0.103	0.045	0.034	2.300	0.021	0.015	0.190
	Asian	0.115	0.076	0.021	1.500	0.134	-0.035	0.264
	Under Represented Minorities	0.068	0.058	0.016	1.162	0.245	-0.047	0.182
	Clinical job role	0.244	0.038	0.092	6.470	0.000	0.170	0.318
	Staff	-0.328	0.052	-0.114	-6.285	0.000	-0.431	-0.226
	Trainee	-0.153	0.076	-0.039	-2.009	0.045	-0.301	-0.004
	Has Child(ren)	0.762	0.039	0.287	19.482	0.000	0.685	0.838
Model 4		2.245	0.139		16.180	0.000	1.973	2.517
	Age	-0.009	0.002	-0.077	-4.910	0.000	-0.012	-0.005
	Female	-0.141	0.105	-0.045	-1.349	0.177	-0.347	0.064
	Married	0.101	0.045	0.033	2.279	0.023	0.014	0.189
	Asian	0.117	0.076	0.021	1.538	0.124	-0.032	0.266
	Under Represented Minorities	0.056	0.058	0.013	0.963	0.336	-0.058	0.170
	Clinical job role	0.127	0.090	0.048	1.401	0.161	-0.051	0.304
	Staff	-0.578	0.105	-0.200	-5.494	0.000	-0.784	-0.372
	Trainee	-0.326	0.131	-0.084	-2.484	0.013	-0.583	-0.069
	Has Child(ren)	0.462	0.076	0.174	6.076	0.000	0.313	0.611

© 2021 Delaney RK et al. *JAMA Network Open.*

	Gender by Have Child	0.401	0.086	0.146	4.665	0.000	0.233	0.570
	Gender by Staff	0.222	0.108	0.083	2.062	0.039	0.011	0.433
	Gender by Trainee	0.307	0.140	0.066	2.196	0.028	0.033	0.581
	Clinical job by Staff	0.190	0.100	0.069	1.895	0.058	-0.007	0.386
	Clinical job by Trainee	-0.170	0.141	-0.025	-1.210	0.226	-0.447	0.106

© 2021 Delaney RK et al. *JAMA Network Open.*

eTable 3. Full Regression Model Predicting Participants' Reported Work Productivity (N=4634)

**Model Summary**									
Model	R	R Square	Adjusted R Square	Std. Error of the Estimate	Change Statistics				
					R Square Change	F Change	df1	df2	Sig. F Change
1	.144[a]	0.021	0.020	1.098	0.021	19.670	5	4628	0.000
2	.265[b]	0.070	0.068	1.070	0.049	81.725	3	4625	0.000
3	.283[c]	0.080	0.078	1.064	0.010	50.420	1	4624	0.000
4	.294[d]	0.087	0.084	1.061	0.006	6.535	5	4619	0.000

**ANOVA[a]**						
		Sum of Squares	df	Mean Square	F	Sig.
Model 1	Regression	118.519	5	23.704	19.670	.000[b]
	Residual	5576.997	4628	1.205		
	Total	5695.516	4633			
Model 2	Regression	399.276	8	49.910	43.584	.000[c]
	Residual	5296.239	4625	1.145		
	Total	5695.516	4633			
Model 3	Regression	456.404	9	50.712	44.758	.000[d]
	Residual	5239.112	4624	1.133		
	Total	5695.516	4633			
Model 4	Regression	493.206	14	35.229	31.279	.000[e]
	Residual	5202.310	4619	1.126		
	Total	5695.516	4633			

© 2021 Delaney RK et al. *JAMA Network Open.*

**Coefficients[a]**								
		Unstandardized Coefficients		Standardized Coefficients	t	Sig.	95.0% Confidence Interval for B	
		B	Std. Error	Beta			Lower Bound	Upper Bound
Model 1		3.592	.071		50.266	.000	3.451	3.732
	Age	-.011	.001	-.111	-7.533	.000	-.014	-.008
	Female	-.159	.038	-.061	-4.166	.000	-.234	-.084
	Married	.093	.038	.036	2.464	.014	.019	.167
	Asian	.187	.067	.040	2.767	.006	.054	.319
	Under Represented Minorities	-.173	.051	-.050	-3.374	.001	-.273	-.072
Model 2		3.733	.095		39.184	.000	3.546	3.920
	Age	-.008	.002	-.087	-5.465	.000	-.011	-.005
	Female	-.059	.038	-.023	-1.572	.116	-.134	.015
	Married	.075	.037	.030	2.033	.042	.003	.148
	Asian	.109	.066	.024	1.654	.098	-.020	.239
	Under Represented Minorities	-.105	.050	-.030	-2.093	.036	-.203	-.007
	Clinical job role	.093	.032	.042	2.849	.004	.029	.156
	Staff	-.515	.045	-.213	-11.444	.000	-.603	-.427
	Trainee	.075	.065	.023	1.160	.246	-.052	.202
Model 3		3.555	.098		36.266	.000	3.363	3.747

© 2021 Delaney RK et al. *JAMA Network Open.*

	Age	-.006	.002	-.063	-3.938	.000	-.009	-.003
	Female	-.054	.038	-.021	-1.426	.154	-.127	.020
	Married	.000	.038	.000	-.007	.994	-.075	.075
	Asian	.106	.066	.023	1.612	.107	-.023	.235
	Under Represented Minorities	-.118	.050	-.034	-2.366	.018	-.216	-.020
	Clinical job role	.085	.032	.038	2.641	.008	.022	.149
	Staff	-.489	.045	-.203	-10.889	.000	-.577	-.401
	Trainee	.156	.065	.048	2.386	.017	.028	.284
	Has Child(ren)	.238	.034	.107	7.101	.000	.173	.304
Model 4		3.779	.119		31.732	.000	3.545	4.012
	Age	-.006	.002	-.064	-3.989	.000	-.009	-.003
	Female	-.095	.090	-.036	-1.054	.292	-.271	.082
	Married	-.001	.038	.000	-.033	.974	-.076	.074
	Asian	.092	.066	.020	1.406	.160	-.036	.221
	Under Represented Minorities	-.119	.050	-.034	-2.390	.017	-.217	-.021
	Clinical job role	-.259	.078	-.117	-3.331	.001	-.412	-.107
	Staff	-.803	.090	-.333	-8.908	.000	-.980	-.626
	Trainee	-.105	.113	-.032	-.927	.354	-.326	.117
	Has Child(ren)	.300	.065	.135	4.598	.000	.172	.428
	Gender by Have Child	-.073	.074	-.032	-.987	.323	-.218	.072

© 2021 Delaney RK et al. *JAMA Network Open.*

	Gender by Staff	.088	.092	.039	.947	.344	-.094	.269
	Gender by Trainee	.153	.120	.039	1.272	.203	-.083	.389
	Clinical job by Staff	.447	.086	.195	5.189	.000	.278	.616
	Clinical job by Trainee	.208	.121	.037	1.721	.085	-.029	.446

© 2021 Delaney RK et al. *JAMA Network Open.*

eTable 4. Full Regression Model Predicting Participants' Reported Concern about COVID-19's Impact on Career Development (N=4657)

**Model Summary**									
Model	R	R Square	Adjusted R Square	Std. Error of the Estimate	Change Statistics				
					R Square Change	F Change	df1	df2	Sig. F Change
1	.281[a]	0.079	0.078	1.214	0.079	79.882	5	4651	0.000
2	.302[b]	0.091	0.090	1.207	0.012	21.169	3	4648	0.000
3	.324[c]	0.105	0.104	1.197	0.014	71.668	1	4647	0.000
4	.333[d]	0.111	0.108	1.195	0.005	5.558	5	4642	0.000

**ANOVA[a]**						
		Sum of Squares	df	Mean Square	F	Sig.
Model 1	Regression	589.007	5	117.801	79.882	.000[b]
	Residual	6858.833	4651	1.475		
	Total	7447.840	4656			
Model 2	Regression	681.460	8	85.182	58.514	.000[c]
	Residual	6766.380	4648	1.456		
	Total	7447.840	4656			
Model 3	Regression	784.228	9	87.136	60.766	.000[d]
	Residual	6663.612	4647	1.434		
	Total	7447.840	4656			
Model 4	Regression	823.882	14	58.849	41.241	.000[e]
	Residual	6623.958	4642	1.427		
	Total	7447.840	4656			

© 2021 Delaney RK et al. *JAMA Network Open.*

**Coefficients**[a]								
		Unstandardized Coefficients		Standardized Coefficients	t	Sig.	95.0% Confidence Interval for B	
		B	Std. Error	Beta			Lower Bound	Upper Bound
Model 1		3.836	0.079		48.590	0.000	3.681	3.991
	Age	-0.029	0.002	-0.267	-18.666	0.000	-0.033	-0.026
	Female	-0.126	0.042	-0.042	-2.984	0.003	-0.208	-0.043
	Married	-0.016	0.042	-0.006	-0.387	0.699	-0.098	0.065
	Asian	0.244	0.074	0.046	3.289	0.001	0.099	0.390
	Under Represented Minorities	0.133	0.057	0.033	2.354	0.019	0.022	0.244
Model 2		4.097	0.107		38.218	0.000	3.887	4.307
	Age	-0.030	0.002	-0.270	-17.237	0.000	-0.033	-0.026
	Female	-0.064	0.043	-0.022	-1.505	0.132	-0.147	0.019
	Married	-0.033	0.042	-0.011	-0.801	0.423	-0.115	0.048
	Asian	0.188	0.074	0.036	2.536	0.011	0.043	0.334
	Under Represented Minorities	0.174	0.057	0.044	3.073	0.002	0.063	0.285
	Clinical job role	-0.100	0.037	-0.040	-2.746	0.006	-0.172	-0.029
	Staff	-0.320	0.051	-0.116	-6.329	0.000	-0.419	-0.221
	Trainee	-0.071	0.073	-0.019	-0.972	0.331	-0.213	0.072
Model 3		3.859	0.110		35.071	0.000	3.644	4.075

© 2021 Delaney RK et al. *JAMA Network Open.*

	Age	-0.027	0.002	-0.243	-15.306	0.000	-0.030	-0.023
	Female	-0.056	0.042	-0.019	-1.328	0.184	-0.139	0.027
	Married	-0.134	0.043	-0.046	-3.115	0.002	-0.218	-0.050
	Asian	0.183	0.074	0.035	2.482	0.013	0.038	0.327
	Under Represented Minorities	0.157	0.056	0.039	2.789	0.005	0.047	0.267
	Clinical job role	-0.110	0.036	-0.044	-3.042	0.002	-0.181	-0.039
	Staff	-0.285	0.050	-0.104	-5.670	0.000	-0.384	-0.187
	Trainee	0.037	0.073	0.010	0.503	0.615	-0.107	0.180
	Has Child(ren)	0.319	0.038	0.126	8.466	0.000	0.245	0.392
Model 4		3.807	0.134		28.453	0.000	3.544	4.069
	Age	-0.027	0.002	-0.241	-15.216	0.000	-0.030	-0.023
	Female	0.151	0.101	0.051	1.498	0.134	-0.047	0.349
	Married	-0.132	0.043	-0.045	-3.080	0.002	-0.216	-0.048
	Asian	0.178	0.074	0.034	2.424	0.015	0.034	0.322
	Under Represented Minorities	0.160	0.056	0.040	2.851	0.004	0.050	0.270
	Clinical job role	-0.272	0.087	-0.107	-3.119	0.002	-0.443	-0.101
	Staff	-0.163	0.101	-0.059	-1.605	0.108	-0.361	0.036
	Trainee	-0.188	0.126	-0.051	-1.490	0.136	-0.436	0.059
	Has Child(ren)	0.381	0.073	0.150	5.191	0.000	0.237	0.524
	Gender by Have Child	-0.076	0.083	-0.029	-0.922	0.356	-0.239	0.086

© 2021 Delaney RK et al. *JAMA Network Open.*

	Gender by Staff	-0.323	0.104	-0.127	-3.115	0.002	-0.526	-0.120
	Gender by Trainee	0.155	0.135	0.035	1.153	0.249	-0.109	0.420
	Clinical job by Staff	0.189	0.097	0.072	1.959	0.050	0.000	0.378
	Clinical job by Trainee	0.249	0.136	0.039	1.835	0.067	-0.017	0.516

© 2021 Delaney RK et al. *JAMA Network Open.*
